# The *Mycobacterium tuberculosis* Drugome and Its Polypharmacological Implications

**DOI:** 10.1371/journal.pcbi.1000976

**Published:** 2010-11-04

**Authors:** Sarah L. Kinnings, Li Xie, Kingston H. Fung, Richard M. Jackson, Lei Xie, Philip E. Bourne

**Affiliations:** 1 Institute of Molecular and Cellular Biology and Astbury Centre for Structural Molecular Biology, University of Leeds, Leeds, United Kingdom; 2 San Diego Supercomputer Center, University of California, San Diego, La Jolla, California, United States of America; 3 Skaggs School of Pharmacy and Pharmaceutical Sciences, University of California, San Diego, La Jolla, California, United States of America; 4 Bioinformatics Program, University of California, San Diego, La Jolla, California, United States of America; National Cancer Institute, United States of America and Tel Aviv University, Israel

## Abstract

We report a computational approach that integrates structural bioinformatics, molecular modelling and systems biology to construct a drug-target network on a structural proteome-wide scale. The approach has been applied to the genome of *Mycobacterium tuberculosis (M.tb)*, the causative agent of one of today's most widely spread infectious diseases. The resulting drug-target interaction network for all structurally characterized approved drugs bound to putative *M.tb* receptors, we refer to as the ‘TB-drugome’. The TB-drugome reveals that approximately one-third of the drugs examined have the potential to be repositioned to treat tuberculosis and that many currently unexploited *M.tb* receptors may be chemically druggable and could serve as novel anti-tubercular targets. Furthermore, a detailed analysis of the TB-drugome has shed new light on the controversial issues surrounding drug-target networks [1]–[3]. Indeed, our results support the idea that drug-target networks are inherently modular, and further that any observed randomness is mainly caused by biased target coverage. The TB-drugome (http://funsite.sdsc.edu/drugome/TB) has the potential to be a valuable resource in the development of safe and efficient anti-tubercular drugs. More generally the methodology may be applied to other pathogens of interest with results improving as more of their structural proteomes are determined through the continued efforts of structural biology/genomics.

## Author Summary

The worldwide increase in multi-drug resistant TB poses a great threat to human health and highlights the need to identify new anti-tubercular agents. We have developed a computational strategy to link the structural proteome of *Mycobacterium tuberculosis*, the causative agent of tuberculosis, to all structurally characterized approved drugs, and hence construct a proteome-wide drug-target network – the TB-drugome. The TB-drugome has the potential to be a valuable resource in the development of safe and efficient anti-tubercular drugs. More generally, the proteome-wide and multi-scale view of target and drug space may facilitate a systematic drug discovery process, which concurrently takes into account the disease mechanism and druggability of targets, the drug-likeness and ADMET properties of chemical compounds, and the genetic dispositions of individuals. Ultimately it may help to reduce the high attrition rate in drug development through a better understanding of drug-receptor interactions on a large scale.

## Introduction

The construction and analysis of molecular interaction networks provides a powerful means to understand the complexity of biological systems and to reveal hidden relationships between drugs, genes, proteins, and diseases. In particular, the study of drug-target networks may facilitate an improved understanding of the principles of polypharmacology and hence improved rational drug design [2]. In recent years, several computational methodologies have been developed to predict drug-target networks based on ligand chemistry [4]–[6], phenotypic changes resulting from drug perturbation [7]–[9], or a combination of chemical features of drugs and sequence features of protein targets [10]–[12]. Extensive experimental and computational evaluation has proven that these methods are valuable for drug repurposing and side effect prediction. However, these methods are biased towards known drug-target pairs, which are mainly derived from well-established human target classes such as G-protein coupled receptors (GPCRs), which only cover a small portion of the human proteome. The lack of a broad spectrum of drug-target pairs is more severe in pathogens than it is in human. For example, amongst the 3,999 proteins encoded by the *Mycobacterium tuberculosis* (*M.tb*) genome, only nine (cmaA1, cyp51, embA, embB, embC, folK, InhA, katG and rpoC) have been pharmaceutically investigated [13]. Thus, drug-target networks that are constructed from only existing drug targets are retrospective, and less capable of discovering new druggable targets and predicting off-target profiles of new compounds on a proteome-wide scale. In addition, the incompleteness of drug-target data poses questions as to whether or not the topology of drug-target networks is inherently modular or random [1].

It is important to construct and analyze a proteome-wide drug-target network that includes not only the primary targets, but also all of the potential off-targets of the drugs in the network. Such a network, if available, would provide unparalleled opportunities for mapping a comprehensive drug-target space and understanding the molecular basis of drug efficacy, side- effects and drug resistance, thereby providing the foundation for the rational design of polypharmacological (multi-target) drugs. For anti-infectious drug discovery, where pharmaceutically investigated targets only represent a small portion of the whole pathogen's proteome, it is more challenging to establish a proteome-wide drug-target network. The linkage of drugs to less exploited proteins such as virulence factors, transport proteins and transcription factors will greatly expand the repository of anti-infectious drug targets and provide new solutions for combating multi-drug and extensively drug resistant pathogens, and for repurposing existing drugs for new uses.

Structural bioinformatics provides an alternative and complementary way to extend drug-target networks to less characterized proteins on a proteome-wide scale. The structural coverage of a given pathogen proteome is usually much larger than the pharmaceutical target coverage. In the case of the *M.tb* proteome, there are 284 unique proteins in the RCSB Protein Data Bank (PDB)[14] (as of November 5, 2009), which is more than 30 times the number of existing pharmaceutical targets for *M.tb*. By taking reliable homology models into consideration, it is possible to increase the structural coverage of the *M.tb* proteome to approximately 43%. By taking advantage of this structural information, we have developed an integrated structural bioinformatics, molecular modelling and systems biology method to construct and analyze a drug-target interaction network, to discover novel druggable targets, and to propose new drug repositioning strategies. Our method is based on the comparison of the binding sites of existing drugs approved for human use against the entire structural proteome of the pathogen under investigation, in order to relate these drugs to new targets. For each identified drug-target pair, the atomic details of the interaction are studied using protein-ligand docking. If the protein is in a metabolic network model, the phenotype change resulting from the drug perturbation is further investigated using flux balance analysis (FBA) of the metabolic network. This strategy has been applied to study several selected drug targets, and proven, both computationally and experimentally, to be a useful tool in drug repositioning [15], side effect prediction [16], [17], and polypharmacological target discovery [18]. In this paper, we extend this methodology to the construction of a proteome-wide drug-target network. Compared with existing methods that are either ligand or target centric, our method provides a framework to correlate the molecular basis of protein-ligand interactions to the systemic behavior of organisms. The proteome-wide and multi-scale view of target and drug space may shed new light on unsolved issues related to drug-target networks, and facilitate a systematic drug discovery process, which concurrently takes into account the disease mechanism and druggability of targets, the drug-likeness and ADMET properties of chemical compounds, and the genetic dispositions of individuals. Ultimately it may help to reduce the high attrition rate during drug discovery and development.

The continuing emergence of *M.tb* strains resistant to all existing, affordable drug treatments means that the development of novel, effective and inexpensive drugs is an urgent priority. However, conventional drug discovery is a time-consuming and expensive process that is poorly equipped in the battle against tuberculosis. In this study, we apply our integrated approach in constructing the drug-target network of *M.tb*, which we refer to as the ‘TB*-*drugome’. Using the TB-drugome we first attempt to characterize all drug-target interactions (i.e., the polypharmacological space) of the *M.tb* proteome and to shed new light on controversial issues surrounding drug-target networks [**1**]–[**3**]. It has been argued that drug-target networks are similar to random networks, and that the observed modularity in drug-target networks may simply be the result of missing links between drugs and targets [1]. Our results support the idea that drug-target networks are inherently modular, and further that any observed randomness is mainly caused by biased target coverage. Then we introduce a new concept, the target chemical druggability index (TCDI), which we use to determine the chemical druggability and prioritization of a protein as a drug target, and to characterize the potential of a drug as a polypharmacological lead compound. The TB-drugome reveals not only that many existing drugs show the potential to be repositioned to treat tuberculosis, but also that many currently unexploited *M.tb* proteins may be highly druggable and could therefore serve as novel anti-tubercular targets. The TB-drugome is publically available (http://funsite.sdsc.edu/drugome/TB) and has the potential to be a valuable resource for the development of safe and efficient anti-tubercular drugs. Structural biology and structural genomics efforts continue to increase the structural coverage of the *M.tb* proteome [**19**]–[**21**], as well as those of other pathogens. This will improve the robustness of the TB-drugome and facilitate the application of this methodology to other pathogens. We hope that the application of the drugome concept will revitalize our way of thinking about how drug discovery is approached, something which is urgently needed if we wish to succeed in this on-going battle against multi-drug and extensively drug resistant infectious diseases.

## Results

### A drug binding site database

**Figure 1:**
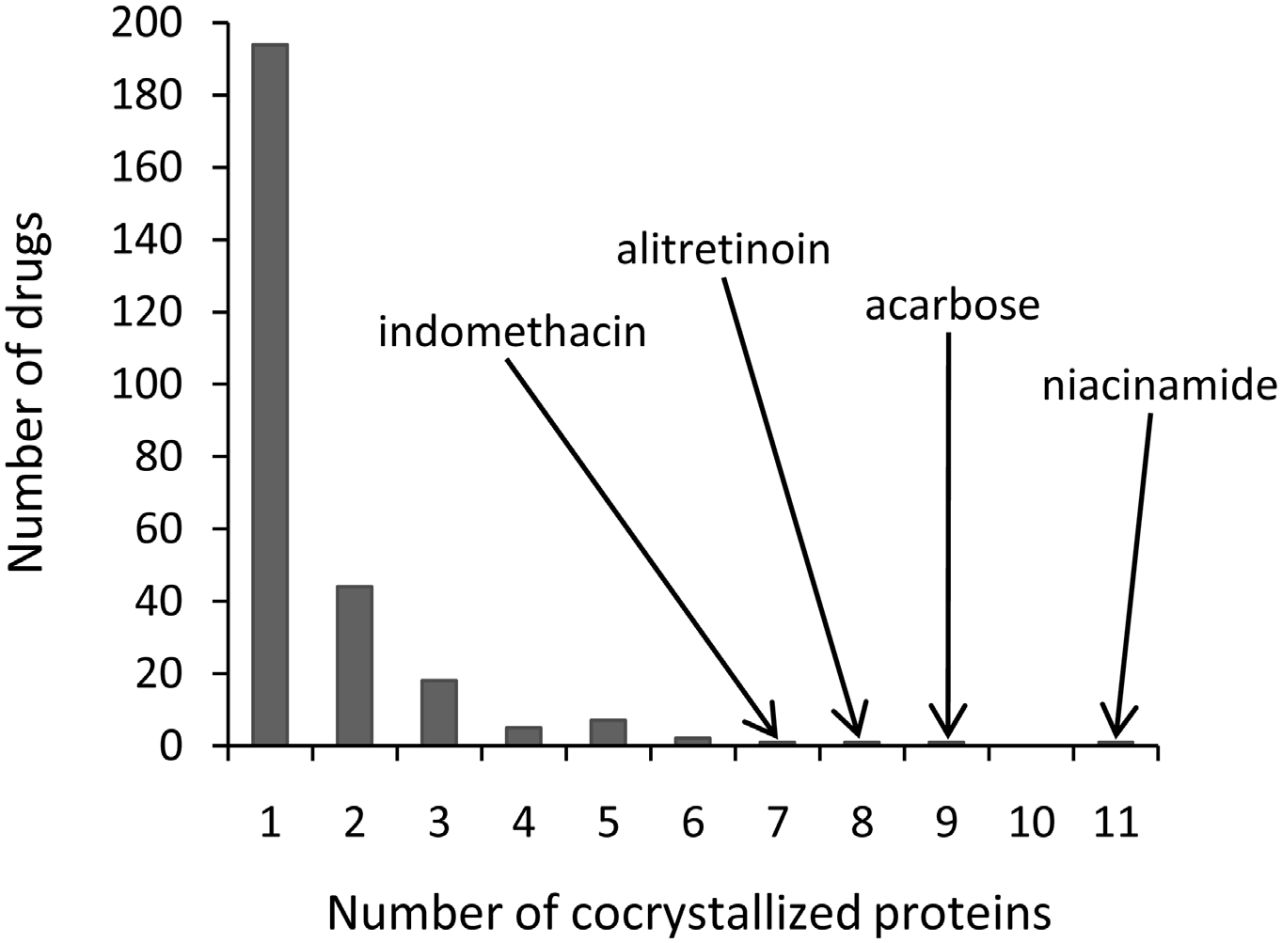
The numbers of unique proteins co-crystallized with approved drugs in the PDB.

A total of 274 different drugs approved for human use in the United States and Europe were identified in the RCSB Protein Data Bank (PDB) [14]. While the majority of these drugs were only co-crystallized with a single protein structure, many drugs were co-crystallized with more than one structure, bringing the total number of drug binding sites in the PDB to 962 (see the Supporting Information, Table S1). Many of these structures were derived from the same protein in different source organisms, and so the number of binding sites per drug is not a good indicator of drug promiscuity. In order to overcome this issue, the number of unique proteins co-crystallized with each drug was determined (Figure 1). While the vast majority of the drugs (194/274) had only been co-crystallized with a single protein, several had been co-crystallized with a number of different proteins, often from completely different folds. With a total of 11, 9, 8 and 7 different binding sites, the drugs niacinamide, acarbose, alitretinoin and indomethacin, respectively, were co-crystallized with the greatest number of different proteins. The distribution of the drug connections of co-crystallized proteins is close to a power-law distribution (Supporting Information, Figure S1). However, most of the proteins are only co-crystallized with a single drug. Only five proteins are co-crystallized with two drugs, and no proteins are co-crystallized with more than two drugs. It is not clear whether or not target connections in the PDB are scale-free.

### TB-drugome: A reliable and unbiased protein-drug interaction network

**Figure 2:**
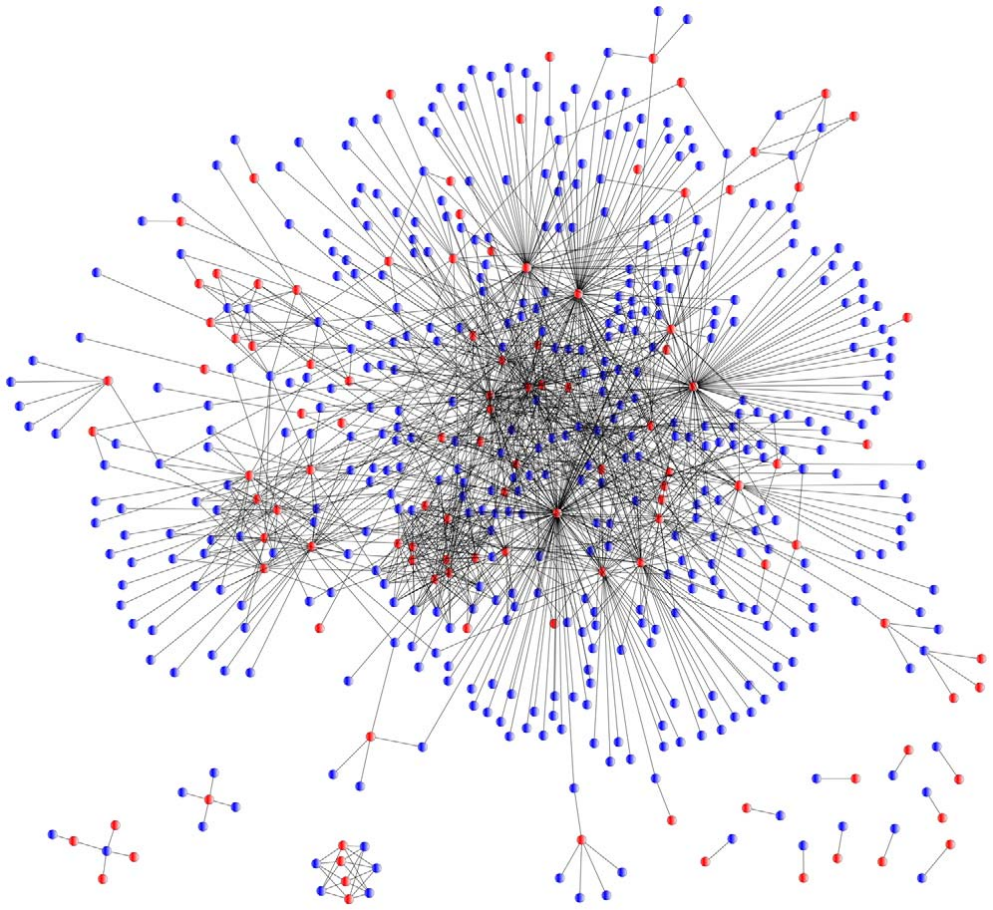
A protein-drug interaction network to illustrate similarities between the binding sites of *M.tb* proteins (blue), and binding sites containing approved drugs (red). A SMAP *P*-value threshold of 1.0e-5 was used.

The TB-drugome, a structural proteome-wide drug-target network of *M.tb*, was constructed by associating the putative ligand binding sites of *M.tb* proteins with the known binding sites of approved drugs for which structural information about the target was available. The premise is that two entirely unrelated proteins can bind similar ligands if they share similar ligand binding sites. In this way, a *M.tb* protein can be connected to a drug through the drug's target, irrespective of whether that protein target is from human or another organism. The binding site comparison software SMAP [**22**]–[**24**], was used for this purpose in an all-drug-against-all-target manner (see the Methods section). In a previous benchmark study, SMAP outperformed most of the existing ligand binding site comparison algorithms [22], [24]. Moreover, several predictions from SMAP have been experimentally validated [15], [18], [25]. Thus SMAP has proven a useful tool to identify the off-targets of existing drugs. The resulting TB-drugome network is shown in Figure 2 and consists of *M.tb* proteins (blue circles) connected to drugs (red circles), where a single connection indicates binding site similarity between any of the structures of the connected *M.tb* protein, and any of the binding sites of the connected drug. The TB-drugome is highly connected, indicating that many binding site similarities were observed between *M.tb* proteins and drug targets, even though those proteins had different overall structures.

**Figure 3:**
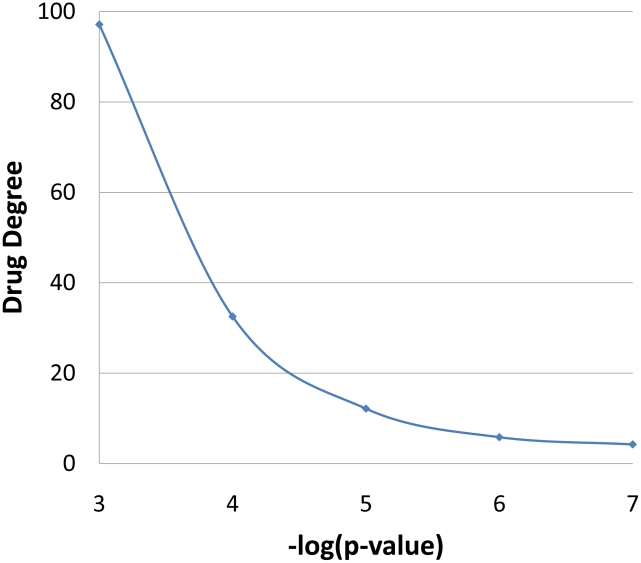
The average number of connections per drug in the TB-drugome against the SMAP *P*-value threshold.

The number of edges in the TB-drugome network depends on the confidence level of the prediction. To determine the SMAP *P*-value threshold that gives a balanced false positive and negative rate in the TB-drugome, the average connectivity of the drugs was plotted against the SMAP *P*-value. A turning point in the curve exists for a SMAP *P*-value of 1.0e-5 (Figure 3), i.e., the connectivity of the drugs changes only slightly with a SMAP *P*-value of less than 1.0e-5, but rapidly increases when the *P*-value is greater than 1.0e-5. The use of a SMAP *P*-value threshold greater than 1.0e-5 will therefore reduce the false negative rate, but dramatically increase the false positive rate when detecting similar ligand binding sites. Thus, a SMAP *P*-value of 1.0e-5 was selected as a threshold for network construction, and was used throughout this study. Based on the previous SMAP benchmark study [22], [24], the false positive rate is approximately 5% when the SMAP *P*-value is close to 1.0e-5. Thus, it is estimated that the false positive rate of connections is approximately 5% in the TB-drugome.

In the TB-drugome, 123 of the 274 drugs are connected to 447 of the 1,730 proteins (284 PDB structures plus 1,446 homology models). Thus, it is estimated that around 40% of these 274 approved drugs, or their associated compound libraries, may be active against around 25% of the *M.tb* structural proteome, greatly expanding the existing anti-tubercular drug-target space. Unlike other drug-target networks [1]–[3], the TB-drugome is not biased towards certain gene families. The largest family in the TB-drugome is cytochrome P450, which consists of 20 proteins, comprising approximately 4.5% of the connected proteins and 1% of all proteins in the TB-drugome, respectively. The average degree of drug connectivity in the TB-drugome is 12.1, which is almost twice the predicted value of 6.3 for drug-target networks [1]. Despite the high degree of drug connectivity, the modularity of the network is maintained, as shown in the next section.

### The TB-drugome is a scale-free and modular network

**Figure 4:**
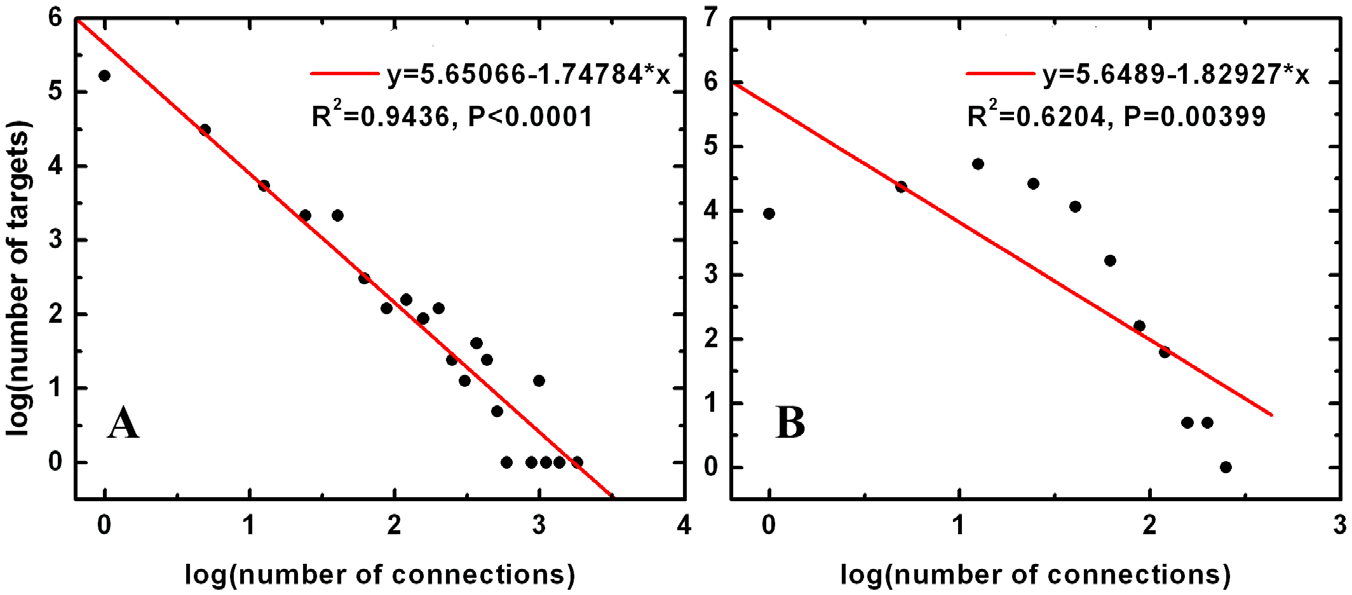
Fitting of the distribution of target connections to a power-law distribution for (A) the TB-drugome and (B) a random network. A SMAP *P*-value threshold of 1.0e-5 was used.

Reliable and unbiased drug-target networks may shed new light on the controversial issues surrounding the underlying topological structure of drug-target networks. It has been argued that drug-target networks are similar to random networks, and that the observed modularity in drug-target networks may simply be the result of missing links between drugs and targets [1]. Topological analysis of the TB-drugome provides evidence for the modularity of drug-target networks. Although the average connectivity of drugs is much higher (Figure 3) than that predicted for a drug-target network in which the targets are pharmaceutically annotated [1], the distribution of target connectivities follows a power-law distribution regardless of *P*-value threshold (Figure 4A and Table 1). That is, most targets have few connections, but a small number of targets are connected to a large number of drugs. This is also true for drug connectivity (Supporting Information, Figure S2 and Table S6). This observation strongly suggests that proteome-scale drug-target networks are not random. This scale-free property is not sensitive to the systematic noise introduced by the increased number of false positive edges that result from an increase in the *P*-value threshold, indicating that the connections between proteins and drugs are not completely random. The connections reflect the underlying evolutionary, geometric and physicochemical relationships between the *M.tb* proteins and the drug targets. In contrast, if the edges in the network were random, this scale-free property would break down (Figure 4B and Table 1). Similarly, the false negative rate also has little effect on the topology of the network since the power-law distribution remains consistent when the number of false negatives is increased as a result of decreasing the *P*-value threshold.

**Table 1:** Fitness of the power law distribution for protein targets in the TB-drugome and corresponding random network at different SMAP *P*-value thresholds.

	TB-drugome				Random Network			
SMAP *P*-value	*k*	log(*a*)	R^2^	*P*-value	*k*	log(*a*)	R^2^	*P*-value
1.0e-3	−1.3645	6.5601	0.8335	<0.0001	0.26237	2.6708	0.0080	0.69172
1.0e-4	−1.6141	6.3413	0.9262	<0.0001	−0.7184	4.7086	0.1395	0.18843
1.0e-5	−1.7478	5.6507	0.9436	<0.0001	−1.8292	5.6489	0.6204	0.00399
1.0e-6	−1.6231	4.6890	0.8321	<0.0001	−1.6799	4.8057	0.6063	0.02281
1.0e-7	−1.4326	3.9845	0.8930	<0.0001	−1.4956	4.1041	0.6271	0.03381

Besides being scale-free, the TB-drugome network is modular, as measured by the clustering coefficient. As shown in Table 2, the clustering coefficients of both the targets and the drugs are almost twice those of the corresponding random networks. Moreover, since there is no significant change in the clustering coefficient when using different SMAP *P-*value thresholds to define the network connectivity, this implies that an underlying architecture exists in the TB-drugome. The modularity of the TB-drugome is also measured by the largest connected component (nLCC). The nLCC values for *M.tb* targets and drugs are 0.93 and 0.84, respectively, compared to nLCC values of 0.97 and 1.0, respectively, for a random network (Supporting Information, Figure S3). This modularity becomes more obvious for high confidence networks that are derived from *P*-value thresholds of 1.0e-6 and 1.0e-7.

**Table 2:** Clustering coefficients for protein targets and drugs in the TB-drugome and corresponding random network at different SMAP *P*-value thresholds.

	Target		Drug	
SMAP *P*-value	TB-drugome	Random network	TB-drugome	Random network
1.0e-4	0.703	0.342	0.783	0.417
1.0e-5	0.663	0.318	0.676	0.351
1.0e-6	0.643	0.339	0.556	0.273
1.0e-7	0.765	0.354	0.786	0.313

Since the 274 structurally characterized drugs only comprise around 20% of all approved drugs, it is interesting to investigate the effects of increasing drug structural coverage on the properties of the network. To address this question, we randomly selected a subset of the 274 structurally characterized drugs to see how the structural coverage of drug-target complexes affects the power-law distribution and the clustering coefficient of the network. Even when the number of drug-target complexes drops to 20% of the total number present in the PDB, there are no significant changes in the network properties of the TB-drugome (Supporting Information, Figure S4, Table S7 and S8). Thus, it is expected that the scale-free properties and modularity observed in the TB-drugome will not be affected by an increase in the number of drug-target complex structures.

One factor that may contribute to the randomness of existing ligand-based drug-target networks is target bias towards several gene families, for instance, G-protein coupled receptors. Proteins in the same gene family tend to cluster together; therefore, if such gene families dominate a network, then a large nLCC value is easily obtained and the power law distribution breaks down. It is to be expected that the topological properties of drug-target networks will change once extended to include the entire proteome. The current incompleteness of drug-target networks is not only due to the missing links between drugs and targets, but also due to the biased and limited coverage of target space. However, as this coverage improves we anticipate that power-law behaviour will be preserved.

### Highly connected proteins are potential chemically druggable targets

To our knowledge, there are currently only nine *M.tb* proteins that have been validated as drug targets; cmaA1 (Rv3392c), cyp51 (Rv0764c), embA (Rv3794), embB (Rv3795), embC (Rv3793), folK (Rv3606c), InhA (Rv1484), katG (Rv1908c) and rpoC (Rv0668) [13]. According to the TB-drugome there are numerous other drug targets yet to be exploited. An important question in drug discovery is whether or not a biologically validated target is able to bind drug-like molecules with high affinity, i.e., whether or not the target is chemically druggable. Although chemical druggability can be predicted from the ligand binding site of a protein [26], there is still a big gap between identifying lead compounds and developing safe drugs. Analysis of the TB-drugome not only provides molecular insights into chemical druggability, but also suggests existing drugs that could either be directly repurposed or act as lead compounds. Here we introduce a new Target Chemical Druggability Index (TCDI), which is orthogonal to biological essentiality, and directly links target and drug space. After the drug target has been biologically validated as an essential gene, the TCDI may be applied to determine if it is a suitable candidate for medicinal chemistry efforts. The TCDI is determined by the number of unique drugs (those with a 2D Tanimoto coefficient to one another of less than 0.75) that are connected to a protein in the TB-drugome. In this way, it is possible to prioritize the chemically druggable targets on a proteome-wide scale. In the TB-drugome, there are 165 proteins with a TCDI of greater than 2. Those proteins with a TCDI of greater than 8 are listed in Table 3. Since most of these proteins have not been pharmaceutically investigated, their propensity to bind drug-like molecules should be determined experimentally.

**Table 3:** Genes in the TB-drugome with a TCDI of greater than 8, and their *in silico, in vitro,* and *in vivo* essentialities, and potential as a drug target.

Gene	TCDI	*in silico* essentiality		*in vitro* Essentiality	*in vivo* Essentiality	Useful Target
		GSMN-TB	iNJ661			
Rv3676	22	X	X		**Essential ** [86]	[29]
inhA (Rv1484)	19	**Essential**	**Essential**			[87]
Rv1264	15	X	Non-essential			[88]
Rv2413c	13	X	X			[34]
ffh (Rv2916c)	11	X	X	**Essential ** [89]		[90]
narL (Rv0844c)	10	X	X			[36], [35]
lprG (Rv1411c)	10	X	X		**Essential ** [91], [92]	[37]
Rv1272c	10	Non-essential	X		**Essential ** [92]	[93]
Rv0856	9	X	X			Function unknown
Rv3644c	9	X	X			[90]
Rv0435c	9	X	X			[90]
proC (Rv0500)	9	Non-essential	**Essential**	**Essential ** [89]		[94]

The gene is marked with an ‘x’ if it was not present in the GMMN-TB or iNJ661 reconstructed metabolic networks.

Although gene essentiality is not necessarily correlated with chemical druggability, it is interesting to investigate whether or not those proteins with a large TCDI are crucial for bacterial survival or virulence. If a gene is both essential and chemically druggable, it will be an ideal target for drug development. The biological roles of these proteins were determined primarily from the literature. Since several of the proteins listed in Table 3 are involved in metabolism, it is possible to investigate the effects of their knockout by carrying out flux balance analysis (FBA) using a proteome-wide network model of *M.tb* metabolism. The GSMN-TB model [27] was selected to simulate *in vivo* conditions, while the iNJ661 model [28] was selected to simulate *in vitro* conditions.

**Figure 5:**
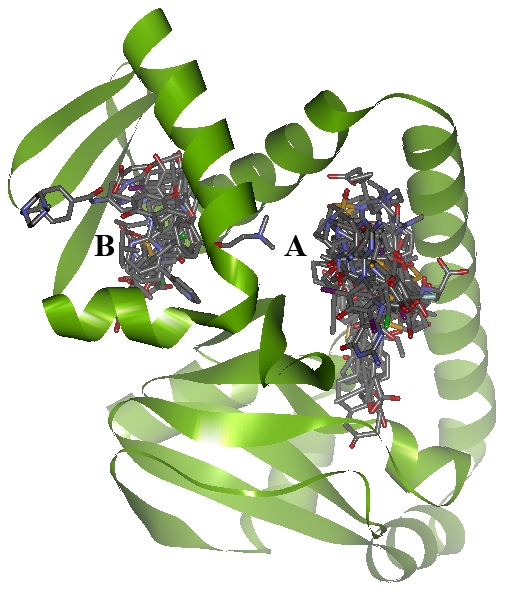
Predicted drug binding sites and poses in *M.tb* CRP/FNR. The AMP binding site is labelled ‘A’. An alternative binding site in the DNA binding domain is labelled ‘B’. The protein is represented as a green ribbon model. Drugs are represented as stick models. Atoms of C, O, N, and S are colored grey, red, blue and yellow, respectively.

Most of the proteins in Table 3 with known functions are essential for bacterial survival, as predicted by metabolic simulation, or validated by experiments. The top ranked protein, Rv3676, encodes the cAMP receptor protein/fumarate and nitrate reductase (CRP/FNR) transcriptional regulator. Members of the CRP/FNR class of transcriptional regulators respond to environmental conditions associated with low oxygen stress and starvation, and may play an important role in reactivating dormant bacilli. The importance of the *M.tb* CRP/FNR transcriptional regulator has been demonstrated through knockout studies. Indeed, deletion of this gene is known to cause growth defects in laboratory medium, in bone marrow derived macrophages and in a mouse model of tuberculosis [29]. 22 unique drugs are predicted to be potential lead compounds targeting CRP/FNR. As shown in Figure 5, besides the known cAMP binding site (site A), a second binding site (site B) is identified in the DNA binding domain. This finding provides opportunities to design drug conjugates or combination therapies to inhibit this protein. The *M.tb* protein with the second highest TCDI is InhA (enoyl-acyl carrier protein reductase), which is actually the target of the front-line anti-tubercular agent isoniazid [30]. As a pro-drug, the therapeutic effect of isoniazid depends on its conjugation with the NAD co-factor. The development of isoniazid-resistant *M.tb* strains has promoted the discovery of a number of direct inhibitors of InhA [31]. Most of the predicted drug binding sites are located in the substrate binding site of InhA, and therefore serve as potential leads for direct InhA inhibitors. Indeed, the prediction that InhA can be directly inhibited by an existing drug has already been experimentally validated. Both an *in vitro* bacterial growth study and an enzyme kinetic assay supported our previous *in silico* prediction that Comtan, a drug used in the treatment of Parkinson's disease, could potentially be repurposed to target InhA directly [15]. Thus the prediction that InhA is a highly druggable target is supported by existing experimental data, in addition to common clinical practice.

There are a number of *M.tb* proteins that, although not predicted to be essential, may play important roles in the host-pathogen interaction. The protein with the third highest TCDI is Rv1264, a class III adenylyl cyclase which synthesizes cAMP from ATP in response to sensing the mildly acidic pH of the host macrophage phagosome. Biochemical studies of Rv1264 have suggested that it may be crucial for the *M.tb* host-pathogen interaction, thereby highlighting it as another potentially interesting drug target [32]. The predicted drug binding site is located in the dimerization interface of the regulatory domain (Supporting Information, Figure S5). Since dimerization is critical for the function of adenylyl cyclase, it is speculated that the inhibition of its association may disrupt its function [33]. Other proteins that are involved in the host-pathogen interaction include Rv2413c [34], narL [35], [36], and lprG [37]. A new strategy emerging to combat drug resistant pathogens is to target the pathways involved in host-pathogen interactions [38]. The identification of druggable targets that contribute towards pathogenicity (e.g., proteins involved in two-component regulatory systems [39], [40]) and the host-pathogen interface may present new opportunities for the discovery of novel therapeutics effective against tuberculosis.

Several other non-essential genes may contribute to drug resistance mechanisms exhibited by *M.tb*. For example, Rv1272c is an efflux pump that detoxifies antibiotics. Combination therapy using antibiotics mixed with efflux pump inhibitors could therefore be a practical solution for increasing the efficacy of antibiotics [41]. In addition, the TB-drugome may provide clues about the biological roles of proteins with unknown functions. Since Rv0856 is predicted to bind to antibiotics such as minocycline and rifampin, it is possible that this protein is involved in the detoxification of these antibiotics.

### Highly connected drugs are candidates for multi-target therapeutics

The TB-drugome reveals that, of the 274 different drugs investigated, 92 drugs could potentially inhibit more than one *M.tb* protein. This is advantageous both in terms of drug effectiveness and preventing the development of drug resistance. Indeed, large-scale functional genomics studies in model organisms have shown that the vast majority of single-gene knockouts actually exhibit little or no effect on phenotype [42]. The concept of ‘synthetic lethality’ - genes that are not essential individually, but are essential in combination - uncovers a whole new plethora of drug targets that may have been overlooked due to their non-essentiality in individual gene knockout studies. Synthetic lethality explains the success of several multi-target anti-infectives that have been discovered serendipitously over the years, including D-cycloserine, beta-lactam antibiotics, fosfomycin and fluoroquinolone antibiotics [43]. Furthermore, inhibition of two or more proteins that are essential individually is advantageous from a drug resistance perspective. Indeed, while pathogens are able to rapidly acquire resistance to single target agents through mutations in the target protein, it is much more difficult to acquire resistance to multiple target agents, since a mutation in one of the essential target proteins would not confer any selective advantage over the wildtype [44].

Some drugs in the TB-drugome have the potential to inhibit a large number of different *M.tb* proteins simultaneously. It is important to note that there are two types of connections in the TB-drugome; those that involve proteins belonging to the same fold, and those that involve proteins belonging to different folds. The detection of functional relationships between proteins belonging to the same fold is considered to be a trivial task because it can be achieved by simply using conventional sequence and structure comparison tools. It is more interesting and novel to relate proteins across fold space, i.e., when the primary drug target and its off-target(s) do not share similar global structures. Such cross-fold connections constitute around 60% of all connections in the TB-drugome (see Tables S2 and S3 in the Supporting Information for a full list of cross-fold connections in the TB-drugome). The 15 most highly cross-fold connected drugs are listed in Table 4, along with the names of the solved *M.tb* proteins to which they are connected. With 98 cross-fold connections, alitretinoin, a drug used to treat cutaneous lesions in patients with Kaposi's sarcoma, is the most highly connected drug. The solved *M.tb* proteins to which it is connected include bioD, InhA and purN, all of which are predicted to be essential *in vivo* by a metabolic network reconstruction of *M.tb* [27]. With 63 different cross-fold connections, levothyroxine, a drug used to treat hypothyroidism, is the second most highly connected drug. Further investigation revealed that it was the structure of levothyroxine bound in the binding site of serum albumin that was determined to be significantly similar to many of the 63 different *M.tb* binding sites. This is interesting because, as a non-specific binder of steroid hormones and a transport protein for various fatty acids, serum albumin is known to be a highly promiscuous protein [45]. While it is not necessarily a useful result for the purposes of this study, the fact that SMAP is able to detect similarities between the binding site of serum albumin and the binding sites of multiple other proteins at least provides some validation that it is working correctly. Serum albumin also accounts for all 24 connections between the drug propofol and various different *M.tb* proteins. Note that although serum albumin is also listed as an intended target of methotrexate, this drug has not actually been cocrystallized with serum albumin in the PDB, and so this does not account for its high connectivity.

**Table 4:** The 15 most highly connected drugs in the TB-drugome.

Drug	Intended Targets	Total Number of Connections	Connected *M.tb* proteins with Solved Structures
Alitretinoin	Retinoic acid receptor RXR-α, β & γ, retinoic acid receptor α, β & γ-1&2, cellular retinoic acid-binding protein 1&2	98	aroG, **bioD**, bpoC, cyp125, embR, glbN, **InhA**, lppX, nusA, pknE, prcA/prcB, **purN**, Rv1264, Rv3676
Levothyroxine	Transthyretin, thyroid hormone receptor α & β-1, thyroxine-binding globulin, mu-crystallin homolog, serum albumin	63	argR, **bioD**, blaI, ethR, glbN, glbO, kasB, lrpA, nusA, prrA, Rv1264, Rv3676, secA1, thyX
Methotrexate	Dihydrofolate reductase, serum albumin	48	argB, **aroF**, **cmaA2**, cyp121, cyp51, lpd, **mmaA4**, **panC**, Rv3676, TB31.7
Estradiol	Estrogen receptor	38	argB, bphD, cyp121, cysM, **InhA**, mscL, pknB, Rv1264, Rv3676, sigC
Rifampin	DNA-direct RNA polymerase beta chain, orphan nuclear receptor PXR, multidrug resistance protein 1	34	**InhA**, lpdA, lppX, mscL, ptpB, Rv3676
4-hydroxytamoxifen	Estrogen receptor, estrogen receptor β, epoxide hydrolase 2, multidrug resistance protein 1, thymidine phosphorylase	33	argB, cysM, **InhA**, katG, lppX, pknB, pknE, Rv1264, Rv1941, Rv3676
Amantadine	Dopamine receptor D1A&2, matrix protein 2	32	(homology models only)
Raloxifene	Estrogen receptor, estrogen receptor β	28	**deoD**, **InhA**, mbtK, pknB, pknE, prcA/prcB, Rv1264, Rv3676, secA1, sigC
Propofol	Serum albumin, gamma-aminobutyric-acid receptor subunit alpha-1, fatty-acid amide hydrolase	24	clpP, glbN, **InhA**
Indinavir	HIV-1 protease, Gag-Pol polyprotein	23	**InhA**, lpdA
Ritonavir	HIV-1 protease	22	**accD5**, **aroK**, **fabH**, lpdA, **panC**, **serA1**, TB31.7
Darunavir	HIV-1 protease, Gag-Pol polyprotein	22	cyp124, devB, **InhA**, lpdA, **panC**
Lopinavir	HIV-1 protease, Gag-Pol polyprotein, protease	22	lpdA, nrdB, pknG, tpiA
Penicillamine	Caspase-1, Ig kappa chain V-III region GOL	20	groEL, **InhA**, nusA, Rv1264, Rv3676
Nelfinavir	HIV-1 protease	20	**fabH**, pknG, **serA1**

The intended targets of the drugs are given as well as the solved *M.tb* proteins to which they are connected in the network. Those genes that were present in the GSMN-TB metabolic reconstruction are underlined and, of these, those whose knockout resulted in a maximal theoretical growth rate of zero or close to zero have been highlighted in bold. Note that only cross-fold connections are considered here.

The front-line anti-tubercular agent rifampin is listed as the fifth most highly connected drug in Table 4. The structure of its known *M.tb* target, DNA-directed RNA polymerase (rpoC) has not been solved, therefore explaining why it is not listed as a potential target in Table 4. However, a suitable homology model of rpoC was identified in ModBase, based on RNA polymerase from the eubacterium *Thermus thermophilus*. The fact that rifampin has connections with six other solved *M.tb* proteins in Table 4 suggests that it may be mediating some of its anti-tubercular effects through proteins other than its known target, rpoC. A recent study showed that rifampin is able to bind to the NAD binding site of ADP-ribosyl transferase [46], which is ranked highly at 24/962 with a SMAP *P*-value of 4.32e-4. Rifampin is predicted to bind to the NAD and FAD binding sites of InhA and lpdA, respectively. Both of these predictions are supported by the compound association listed in the TDR target database [47]. Since the off-targets of rifampin may be involved in drug metabolism and detoxification, the proteome-wide identification of off-targets may provide molecular insight into the understanding of drug resistance mechanisms.

A literature search of the *M.tb* proteins listed in Table 4 reveals that most of them are potentially novel targets for the development of anti-tubercular therapeutics. For instance, aroF (chorismate synthase), aroG (chorismate mutase) and aroK (shikimate kinase) are attractive targets because they are all involved in the shikimate pathway, which is both essential for the viability of *M.tb*, and absent from humans [48]. LppX is a lipoprotein required for the translocation of complex lipids to the outer membrane, and disruption of the lppX gene has been shown to result in attenuation of virulence of the tubercle bacillus [49]. Another protein that is essential for the pathogenesis and virulence of *M.tb* is the sigma factor sigC, which controls the environment dependent regulation of transcription [50]. A potential target against *M.tb* persistence is the universal stress protein, TB31.7, which is required for the entry of the tubercle bacillus into the chronic phase of infection in the host [51]. These are merely a few examples of the many potentially interesting *M.tb* targets listed in Table 4. Furthermore, there are likely to be many more attractive targets in the form of homology models, which have not been investigated here.

Since many of the genes encoding the *M.tb* proteins listed in Table 4 are involved in metabolism, it is possible to investigate the effects of their knockout using a proteome-scale network model of *M.tb* metabolism. The GSMN-TB model [27] was selected for this purpose due to its ability to simulate *in vivo* conditions. Those genes that were present in the GSMN-TB model, and whose knockout could therefore be simulated, are underlined in Table 4. Those genes whose knockout resulted in a maximal theoretical growth rate of zero or close to zero were considered essential and have been highlighted in bold. All of the drugs in Table 4, with the exception of amantadine and lopinavir, are predicted to potentially inhibit one or more essential metabolic proteins with solved structures. In particular, the anti-HIV therapeutic ritonavir could potentially inhibit a total of five different essential proteins involved in metabolism; accD5 (propionyl-CoA carboxylase), aroK (shikimate kinase), fabH (3-oxoacyl-(acyl carrier protein) synthase III), panC (pantoate—beta-alanine ligase) and serA1 (D-3-phosphoglycerate dehydrogenase). Amantadine has connections to homology models only and so was excluded from this study. Although lopinavir may not inhibit any essential metabolic proteins, some of the proteins that it *could* potentially inhibit may be interesting anti-tubercular targets. For instance, pknG, a eukaryotic-type protein kinase, has been shown to support the survival of mycobacteria in host cells [52], and is required for the intrinsic resistance of mycobacterial species to multiple antibiotics [53]. In addition, the GSMN-TB model was used to simulate multiple gene knockouts and therefore the effect of a single drug inhibiting multiple metabolic proteins simultaneously. For each drug (excluding amantadine and lopinavir), the combined knockout of all metabolic genes listed in Table 4 resulted in zero or close to zero biomass (except for the case of levothyroxine, where combined inhibition of bioD (essential) and thyX (non-essential) resulted in growth). More studies are required to verify this prediction.

### The multi-drug-multi-target space of polypharmacology

**Figure 6:**
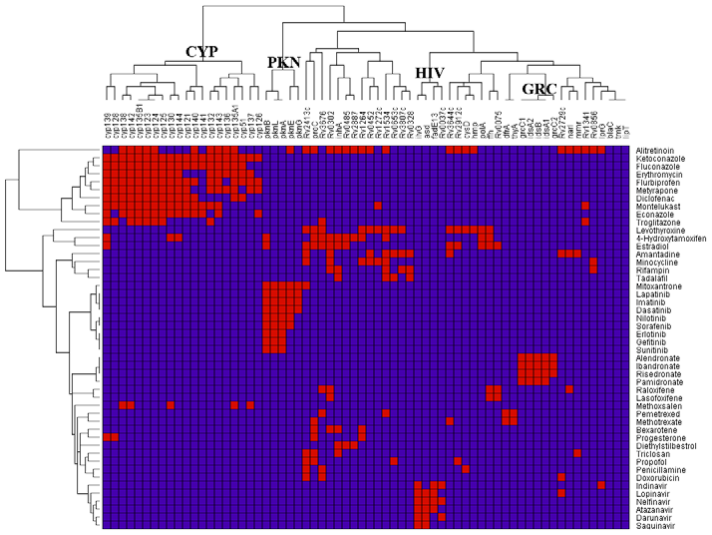
Hierarchal clustering of drug-target binding profiles in the TB-drugome. The grid is colored red if there is a connection between a protein and a drug in the TB-drugome, otherwise, it is colored blue. Each row and column in the matrix corresponds to a binding profile of a drug and a protein, respectively. The three largest clustered gene families are the cytochrome P450s (CYP), protein kinases (PKN), and polyrenyl-diphosphate/polyrenyl synthases (GRC). A new gene cluster (HIV) is predicted to bind to HIV-1 protease inhibitors. For the purpose of clarity, a SMAP *P*-value threshold of 1.0e-6 has been used.

If all members of a set of proteins can bind to the same set of multiple drugs, this set of proteins could provide interesting targets for polypharmacological drugs. Such polypharmacological drug targets can be derived from the TB-drugome. Indeed, several multi-drug-multi-target clusters are distinguishable within the drug-target matrix shown in Figure 6. The three largest clusters are the cytochrome P450s (CYP), protein kinases (PKN), and polyrenyl-diphosphate/polyrenyl synthases (GRC). As promiscuous metabolizing enzymes, the cytochrome P450s bind to multiple drugs, while the protein kinases and polyrenyl-diphosphate/polyrenyl synthases bind human protein kinase inhibitors and farnesyl-diphosphate synthase inhibitors, respectively. Although this result is not surprising, the fact that similar drugs and similar targets are clustered together provides further validation of the TB-drugome.

An interesting cluster is ilvG (acetolactate synthase), asd (aspartate-semialdehyde dehydrogenase), fadE13 (acyl-CoA dehydrogenase) and Rv0037c (MFS-type transporter), all of which are predicted to bind to HIV-1 protease inhibitors. There is a major problem with coincidence of HIV and tuberculosis in sub-Saharan Africa. Indeed, HIV and tuberculosis form a deadly combination, each accelerating the other's progress. Since HIV weakens the immune system, HIV-positive individuals are much more susceptible to developing an active form of tuberculosis and becoming infectious [54]. Co-administration of existing anti-TB and anti-HIV therapeutics is undesirable due to adverse side-effects. Therefore, the finding that an anti-HIV therapeutic can actually be used to treat both HIV and TB simultaneously would be of great interest. It is also worth noting that five of the top 15 most highly connected drugs in the TB-drugome, which are listed in Table 4, are also HIV-1 protease inhibitors.

## Discussion

### A new method to construct a structural proteome-wide drug-target network

All existing drug-target networks have been constructed from annotated drug-target pairs or predicted based on the chemical properties of the ligands from these drug-target pairs. As a result they only include the limited number of human drug targets that have been pharmaceutically investigated, i.e., a small, highly biased subset of the human proteome. The lack of a broad spectrum of drug-target pairs is more severe in pathogens than it is in human. For example, among the 3,999 proteins encoded in the *M.tb* genome, only nine proteins (cmaA1, cyp51, embA, embB, embC, folK, InhA, katG and rpoC) have been pharmaceutically investigated [13]. Conventional methods can only build a drug-target network based on these nine proteins and their associated ligands. Thus, they cannot generate a comprehensive drug-target network like the TB-drugome. The chemical systems biology strategy applied in this paper provides a complementary approach to constructing a structural proteome-wide drug-target network. To our knowledge, the TB-drugome is the first drug-target network that covers this many proteins in the TB structural proteome and all drugs that have been structurally characterized. The TB-drugome includes 50 times more proteins than the existing TB targets, and more than 100 drugs that have not been investigated for tuberculosis treatment. Compared with existing methods that require information about drug-target pairs, one of the unique features of the TB-drugome is that the relationship between two proteins can be established by their ligand binding site similarity, independent of their associated ligands. This feature not only greatly extends target coverage to those proteins with unknown or less characterized ligands, but also includes drugs that may not necessarily be used to target TB proteins directly. Thus, the resulting network is more complete and less biased. Since the TB-drugome includes a large number of poorly characterized or uncharacterized proteins, it may provide greater insight into the progressive drug discovery process than existing drug-target networks. Indeed, it may aid the discovery of novel druggable targets that have not been explored previously, guide medicinal chemists to design compounds with desirable specificity to avoid unwanted side effects, and promote the rational design of polypharmacological drugs by selecting multiple suitable targets. Coincident with recent efforts involving screening compound libraries of existing human drug targets to treat anti-infectious diseases [25], [55], [56], our method will be particularly useful in genome-wide compound profiling, lead generation from existing drug-like molecules, and identifying the molecular targets of active compounds. It is not feasible to achieve such goals using existing drug-target networks in cases where the actual molecular targets or their ligands are unknown.

### Towards a proteome-wide multi-scale protein-drug interaction network

Notwithstanding, there are major limitations in the methodologies applied in this study. Firstly, the structural coverage of the *M.tb* proteome is limited. Currently only 7.2% of *M.tb* proteins have solved structures in the PDB. The use of reliable homology models increases the structural coverage to around 43%. However, each homology model consists of only a single chain rather than the entire biological unit, which could be a multi-polypeptide chain complex. As a result interesting binding sites located in the interface between the chains may be missed. Similarly, only around 20% of all drugs approved for human use have actually been solved with a protein target structure in the PDB. Coverage of drug space can be increased by using crystal structures or homology models of proteins that are known targets of approved drugs, but for which there are no structures with the drugs bound. For instance, the additional inclusion of homology models of GPCRs would double the number of targets.

Two proteins may bind to similar ligands even though their binding pockets may have varied geometrical and physicochemical properties. Such proteins may be sequence homologues, have similar structures, or belong to entirely different folds. For the first two scenarios, SMAP is more sensitive than conventional sequence and structural comparison methods in detecting ligand binding site similarity [24]. For the third scenario, SMAP takes into account residue mutations and geometrical variances within the binding site, therefore making it a sensitive algorithm for ligand binding site similarity searches. However, a fraction of true positives may still be missed in all three scenarios. Despite the existence of false negatives in the drug-target network, the TB-drugome has generated abundant testable hypotheses. From the point of view of real-life applications, it may be more important to reduce the false positive rate than to reduce the false negative rate. The further construction of reliable proteome-wide drug-target networks will benefit from the integration of diverse techniques such as ligand-centric methods [4], [57] and omics data such as gene expression profiles in response to drugs [9]. The integration of multiple data resources will not only increase the coverage of the network, but also the confidence of the predictions made, through the use of consensus results.

Aside from the false negatives that result from the limited structural coverage of the *M.tb* proteome and the completely different ligand binding poses, ligand binding site similarity is necessary but not sufficient to determine the cross-reactivity between two proteins for a specific ligand. The chemical nature of the ligand also determines off-target binding. Although off-target predictions based on similar ligand binding sites are invaluable for the progressive design of selective or polypharmacological drugs, they may result in false positive connections between proteins and existing drugs. Thus, the TCDI may be not correlated with the docking scores. The direct assessment of protein-drug interactions using protein-ligand docking may solve part of this problem, but success is not guaranteed due to the inaccuracy of docking scoring functions. While free energy calculations based on molecular dynamics simulations may improve the prediction of protein-ligand interactions, they are computationally intensive and currently impractical on a proteome-wide scale. It remains a significant challenge to develop new methodologies for accurate and efficient protein-ligand docking and free energy calculation for the prediction of drug off-targets on a proteome-wide scale.

### Molecular basis for the topological structure of drug-target networks

It has been argued that drug-target networks are not modular but random [1]. Drug-target networks constructed by linking all drug-target pairs from annotated chemical libraries or computationally predicted results are limited and biased. Mestres *et al.* discovered that the topologies of drug-target networks are implicitly dependent on drug properties and target families [58]. Consequently, given the biased coverage of target families, the topological properties observed in drug-target networks may not necessarily reflect the inherent properties observed in proteome-wide protein-ligand interaction networks. Here we suggest that modularity does exist in our structural proteome-wide drug-target network, and that it follows a power-law distribution. Any observed randomness appears to result from the biased coverage of drug targets. The power-law distribution has been observed in many biological networks including protein-protein interaction networks [59] and metabolic networks [60], [61]. Recently, it has been found that interaction networks between proteins and their endogenous ligands follow a power-law distribution [62]. Such connectivity distributions also appear in other man-made networks, such as the World Wide Web and social networks. The preferential attachment principle [63], [64], which has been tested in social networks, can be applied to biological networks [62], [65] according to the evolutionary history of ligands and proteins. These studies have shown that evolutionarily ancient ligands and proteins tend to have more connections. It follows that the local structures of the binding site and the core fragments of the ligand are more conserved than global structures and sequences [66].

In the case of protein-ligand interaction networks, the structural basis behind their power-law distribution and scale-free nature could be the modularity of protein-ligand binding sites, the modular arrangement of chemical fragments [67], [68], and the flexibility of both ligand [69] and protein structures [70]. By studying the characterizing descriptors for ligands and small molecules, Ji *et al.* found that polar molecular surface area, H-bond donor counts, H-bond acceptor counts and partition coefficients are key factors that can be used to discriminate hub ligands from others [62].

### A new concept to determine a target's chemical druggability

There are two aspects of target druggability; biological and chemical. From a biological point of view, druggability is conventionally based upon multiple criteria such as gene essentiality, conservation across kingdoms, protein-protein interactions, redundancy among pathways, endogenous metabolite distributions, and coupling between metabolic, regulatory and signalling pathways. However, a biologically druggable essential gene is not necessarily chemically druggable because it may be difficult to design a drug-like molecule to bind it with high affinity and specificity. Thus, biologically validated drug targets need to be linked to their chemical space as early as possible in order to determine their chemical druggability. Although chemical druggability can be predicted from the ligand binding site of a protein [26], there is still a big gap between identifying lead compounds and developing safe drugs. The Target Chemical Druggability Index (TCDI) proposed here is intended to bridge the target validation process and medicinal chemistry efforts to select targets that are both essential (as determined from other resources or methodologies) and appropriate for use in the design of drug-like molecules.

If the functional site of a single protein is connected to, and could therefore potentially be inhibited by, one or more approved drugs, this is a strong indication that this protein may be chemically druggable. Moreover, if a protein has a high TCDI, this implies that any new ligand found will likely occupy the chemically constrained space of approved drugs, as opposed to the essentially unlimited chemical space, and this could benefit drug discovery in many ways. Firstly, it could narrow down the infinite chemical space needed for high-throughput screening to identify lead compounds. Secondly, it provides information about the ligand binding site, which is critical for rational drug design. Thirdly, it may reduce medicinal chemistry efforts to optimize the lead compound as a drug candidate. Finally, and perhaps most critical in this new era of drug discovery, it offers more opportunities to design polypharmacological drugs, which may not only improve drug efficacy and combat drug resistance, but also minimize human side effects. By taking gene essentiality data, chemical druggability information, ligand binding site information, and the ligand coverage of drug space into account simultaneously, the significant time and costs associated with anti-infectious drug discovery and development could be significantly reduced.

A search of the TDR target database [47] reveals that there are no chemical compounds associated with any *M.tb* proteins with a high TCDI other than InhA. Thus, the TB-drugome provides abundant testable hypotheses for the development of new anti-tubercular therapeutics. It is expected that the discovery of a drug candidate by the targeted screening of these drugs will require a fraction of the time and costs associated with conventional high-throughput screening. Even if a drug shows weak activity in an initial assay, the assay can be extended to include the large number of analogues of that drug that have already been synthesized and tested. In this way, it may be possible to discover a potent compound that weakly inhibits the primary drug target, but strongly binds to the *M.tb* target. Such a strategy has been successfully applied to repurpose a library of protein kinase inhibitors to target bacterial biotin carboxylase [25].

### A concurrent versus linear drug discovery process

Conventional drug discovery and development proceeds as a linear process from target identification and validation, to lead discovery and optimization, to preclinical and clinical trials. It is estimated that more than 90% of drug candidates fail during the late stages of drug development, mainly due to poor efficacy or safety [71]. If information were available about disease mechanisms, target druggability, the chemical space of the target, the pharmacokinetics and dynamic properties of drug candidates, and their potential off-targets that may result in unwanted side effects (or sometimes a desirable therapeutic effect), then their consideration in drug development would help to optimize resource allocation and improve productivity in the pharmaceutical industry [72]. Proteome-wide multi-scale drug-target interaction networks help here by providing a resource to unify disease, target, and chemical space, thereby allowing the simultaneous assessment of target essentiality, target druggability, drug design feasibility, chemical availability, compound toxicity, and individual drug response.

In the context of anti-infectious drug discovery, network analysis can be used to identify critical nodes in molecular networks which could represent novel drug targets [28] as illustrated here. Moreover, it is believed that druggability and essentiality are best assessed at the binding site level rather than the global sequence or structural level [43]. Thus, the integration of ligand binding site characterization with systems biology is critical for target identification and prioritization. Even if druggability can be assessed by analyzing the ligand binding site of the target, there is still a huge gap between identifying hit compounds and producing drug candidates. Moreover, the drug candidate may not be safe for human use due to undesirable ADME properties or unwanted off-target effects. By bridging target and drug space, drug-target interaction networks provide invaluable information about the use of existing drugs as lead compounds. In an ideal situation, the drug can be repositioned directly to target the intended target in the pathogen, hence promising a solution to reduce both the time and costs associated with drug development [73]. Since the drugs have already been approved for human use, it is possible to bypass toxicological and pharmacokinetic assessments, which together contribute approximately 40% of the overall cost of bringing a new drug to market. Newly identified drug indications can be evaluated relatively quickly in phase II clinical trials, which typically only take two years and cost around $17 million [74], [75].

### Conclusions

The continuing emergence of *M.tb* strains that are resistant to all existing affordable drug treatments means that the development of novel, effective and inexpensive drugs is an urgent priority [15], [76]. However, current drug discovery methods appear inadequate in the battle against infectious diseases such as tuberculosis [74]. Drug repositioning provides a promising solution to reduce both the time and costs associated with drug development [73]. We have developed a computational approach to compare the binding sites of a subset of existing drugs approved for human use against the entire *M.tb* structural proteome. In this way, it is possible to identify putative new targets of existing drugs within the *M.tb* proteome, providing the basis for their repositioning to treat tuberculosis. Our drug-target interaction network, the TB-drugome, revealed not only that many existing drugs show the potential to be repositioned to treat tuberculosis, but also that some drugs show the potential to be multi-target inhibitors. This is beneficial since multi-target therapy is thought to be more effective than single-target therapy when treating infectious diseases [77]. In addition, the TB-drugome suggests that a large number of *M.tb* proteins are potentially druggable and could therefore serve as novel drug targets in the fight against tuberculosis. We provide the TB-drugome (http://funsite.sdsc.edu/drugome/TB) for analysis by others.

## Methods

### Structural coverage of the *M.tb* proteome

There are 3,996 proteins in the *M.tb* proteome, 284 of which have solved structures in the RCSB PDB (November 5, 2009). Although this approximates to only 7.2% structural coverage of the *M.tb* proteome, it is worth noting that there is likely to be a strong bias towards those targets being relevant to drug discovery. There are multiple structures available for many of these proteins (i.e., a single protein may have been solved with a number of different ligands), bringing the total of solved *M.tb* structures to 749 (November 5, 2009) (see Table S4 in the Supporting Information for further details). It was decided that all 749 of these structures should be used in this study, since a single protein may exhibit multiple binding modes and such information would be missed if only a single structure was chosen to represent each of the 284 proteins. It is important to note that the whole biological unit, rather than a single chain of each structure was used in the case of experimental structures so as to capture ligand binding sites at the interface between polypeptide chains.

ModBase [78], a database of annotated comparative protein structure models, contains homology models for the entire M.tb proteome. However, since they are derived from an automated pipeline, it is likely that some of these models may contain significant errors. Each model in ModBase has been assigned a score corresponding to its reliability, which is derived from statistical potentials. A model is predicted to be reliable if its model score is greater than 0.7 and its ModPipe Protein Quality Score (MPQS) is greater than 1.1 (http://modbase.compbio.ucsf.edu/modbase/modbase_help.html). By employing these thresholds, it is possible to discard unreliable models. ModBase was found to contain ‘reliable’ homology models for a total of 1,446 unsolved M.tb proteins (see Table S5 in the Supporting Information for further details). Through the additional use of these reliable homology models, the structural coverage of the M.tb proteome was increased to around 43%. However, only a single chain of each homology model was available, rather than the entire biological unit.

### Identification of FDA-approved drug binding sites

Drugs approved for human use in the United States and Europe are listed in the U.S. Food and Drug Administration (FDA) Orange Book (http://www.accessdata.fda.gov/scripts/cder/ob/default.cfm) and by the European Medicines Agency (EMEA) (http://www.emea.europa.eu/htms/human/epar/a.htm), respectively. The names of the active ingredients of these drugs were extracted and mapped to compounds in three databases; PubChem (http://pubchem.ncbi.nlm.nih.gov/), DrugBank [13], [79] (http://www.drugbank.ca/) and ChEBI (http://www.ebi.ac.uk/chebi/). After removing all nutraceuticals and prodrugs, InChI keys were used to map the remaining compounds to protein crystal structures in the PDB. Non-protein crystal structures such as DNA, RNA and ribosomes were excluded. 274 different drugs were identified bound to a total of 962 different protein binding sites (November 30, 2009). A full list of the approved drug binding sites used in this study is provided in the Supporting Information, Table S1.

### Comparison of ligand binding sites using SMAP

Xie *et al*. recently developed the ligand binding site comparison software SMAP [22], which is based on a sequence order independent profile-profile alignment (SOIPPA) algorithm [24]. Firstly, the protein structure is characterized by a geometric potential; a shape descriptor that is analogous to surface electrostatic potential, but which uses a reduced C-alpha only structural representation of the protein. It has been shown that both the location and the boundary of the ligand binding site can be accurately predicted using the geometric potential [23]. The reduced representation of the protein structure makes the algorithm tolerant to protein flexibility and experimental uncertainty; thus SMAP can be applied to low-resolution structures and homology models. Secondly, two protein structures are aligned, independent of sequence order, using a fast, maximum weighted sub-graph (MWSG) algorithm [80], [81]. The MWSG finds the most similar local structures in the spirit of local sequence alignment. Finally, the aligned surface patches are ranked by a scoring function that combines evolutionary, geometric and physical information. The statistical significance of the binding site similarity is then rapidly computed using a unified statistical model derived from an extreme value distribution [22].

The SMAP software was used to compare the binding sites of the 749 *M.tb* protein structures plus 1,446 homology models (a total of 2,195 protein structures) with the 962 binding sites of 274 approved drugs, in an all-against-all manner. While the binding sites of the approved drugs were already defined by the bound ligand, the entire protein surface of each of the 2,195 *M.tb* protein structures was scanned in order to identify alternative binding sites. For each pairwise comparison, a *P*-value representing the significance of the binding site similarity was calculated.

### Comparison of global protein structures using FATCAT

FATCAT (Flexible structure AlignmenT by Chaining Aligned fragment pairs allowing Twists) [82] is a program for the flexible comparison of protein structures. It optimizes the alignment between two structures, whilst minimizing the number of rigid body movements (twists) around pivot points introduced in the reference structure. In addition to the optimal structural alignment, FATCAT reports the statistical significance of the structural similarity, measured as a *P*-value. In order to identify pairs of similar binding sites that were from proteins with dissimilar global structures (i.e., cross-fold connections), the first chain of each PDB file was aligned using FATCAT, and those pairs with a significant *P*-value of less than 0.05 were discarded.

### Visualization of the protein-drug interaction network

yEd Graph Editor from yWorks (http://www.yworks.com/en/products_yed_about.html) was used to visualize the drug-target interaction network. *M.tb* protein names were taken from the NCBI Entrez protein database (http://www.ncbi.nlm.nih.gov/protein), to avoid inconsistencies in the naming of proteins in the PDB.

### Flux balance analysis

GSMN-TB [27], a web-based genome-scale network model of *M.tb* metabolism was used to carry out flux balance analysis (FBA) computations. The GSMN-TB model contains 739 metabolites and 726 genes that are involved in 849 unique reactions. For those *M.tb* genes of interest that were also present in the GSMN-TB model, the single gene knockout tool was used to run essentiality prediction under conditions optimized for *in vivo* growth. If the resulting maximal theoretical growth rate was zero or close to zero, then a gene was predicted to be essential, whereas if it was the same as wildtype (0.050191 mmol/g DW/h), it was predicted to be non-essential. In order to simulate multiple gene knockouts, the reactions in which these genes were involved were constrained by setting their upper and lower bound values to zero. Note that this was only carried out for those reactions that could not be carried out by any other genes, i.e., those that were entirely dependent on the gene of interest.

iNJ661 [28] is another genome-scale metabolic reconstruction of *M.tb* that contains 828 metabolites and 661 genes which are involved in 939 reactions. In order to determine *in vitro* essentiality we used the COBRA Toolbox [83] to perform single gene deletions on the iNJ661 model grown in Middlebrook 7H9 media. Again, genes were predicted to be essential if the maximal theoretical growth rate resulting from their deletion was zero or close to zero.

### Molecular docking using eHiTS

For those pairs of interest, molecular docking was used to predict the binding pose and affinity of the drug molecule to the *M.tb* protein. eHiTS Lightning [84] was selected due to its fast speed, relatively high accuracy and ease of automation for large-scale docking studies. Since SMAP had aligned the drug binding site with the *M.tb* protein binding site, the aligned coordinates of the drug molecule were used to define the search space for docking that drug into the *M.tb* protein. The aligned drug molecule was used as the *clip* file with a default search space of 10Å^3^. As recommended by the manual, the eHiTS accuracy level was set to 6 (default  = 3), in order to increase the accuracy of the predicted binding poses. Following all docking, the binding pose with the lowest estimated binding affinity was selected for further investigation. For those proteins with cofactors (e.g., InhA has an NAD cofactor), the cofactor was added as the last residue in the protein structure prior to docking.

### Network analysis

The drug-target interaction network can be represented as a graph. The number of targets or drugs against their connectivity in the graph can be fitted to a power-law distribution, where: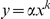 y and x are the number of targets or drugs and their connectivity, respectively, and α and *k* are two fitted parameters.

A protein graph was constructed for the drug-target network. Nodes represented proteins and edges were formed between two protein nodes if they were connected to the same drug. Then the fraction of the largest connected component (nLCC) of the protein was computed by dividing the number of proteins in the largest single linkage cluster by the total number of proteins in the graph. The nLCC values of drugs can be computed in a similar manner.

### Hierarchical clustering of protein and drug binding profiles

Protein and drug binding profiles in the TB-drugome were hierarchically clustered using GenePattern 2.0 [85]. The distance between the profiles was measured using the city block distance.

### Comparison of drug chemical similarity

The 2D fingerprint similarity of drugs was computed using OpenBabel 2.1.1 (http://openbabel.org).

## Supporting Information

Figure S1 Fitting of the distribution of drug connections to a power-law distribution for co-crystallized drug complexes in the PDB.
(0.15 MB DOC)

Figure S2 Fitting of the distribution of drug connections to a power-law distribution for the TB-drugome and a random network.
(1.06 MB DOC)

Figure S3 Fraction of the largest connected component (nLCC) in the network for the TB-drugome and a random network at different SMAP P-value thresholds.
(0.05 MB DOC)

Figure S4 The clustering coefficient of the TB-drugome derived from different fractions of structurally characterized drugs.
(0.07 MB DOC)

Figure S5 Predicted drug binding site and poses in adenylyl cyclase.
(0.75 MB DOC)

Table S1 Information about the approved drug binding sites used in the TB-drugome. This file contains information about the 274 approved drugs that were identified in the PDB. For each drug, its name, PDB ligand code, isomeric SMILES string and known targets are listed, and the PDB codes of the protein structures with which it has been crystallized are given.
(0.09 MB XLS)

Table S2 Cross-fold drug-target pairs in the TB-drugome (for solved M.tb structures only). This file contains a list of the cross-fold drug-target pairs with a SMAP P-value <1.0e-5, for solved M.tb structures only. For each pair, information about the drug and target structures is given, as well as the corresponding SMAP P-value (indicating the significance of the binding site similarity) and eHiTS energy score (from docking the drug into the predicted binding site in the M.tb protein).
(0.08 MB XLS)

Table S3 Cross-fold drug-target pairs in the TB-drugome (for M.tb homology models only). This file contains a list of the cross-fold drug-target pairs with a SMAP P-value <1.0e-5, for homology models of M.tb proteins only. For each pair, information about the drug and target structures is given, as well as the corresponding SMAP P-value (indicating the significance of the binding site similarity) and eHiTS energy score (from docking the drug into the predicted binding site in the M.tb protein).
(0.15 MB XLS)

Table S4 Information about the solved M.tb structures used in the TB-drugome. This file contains information about the M.tb proteins with solved structure(s) in the RCSB PDB that were used in the the TB-drugome. For each protein, the gene name (if available), gene accession number, protein name and corresponding PDB codes are given.
(0.06 MB XLS)

Table S5 Information about the M.tb homology models used in the TB-drugome. This file contains information about the reliable homology models of M.tb proteins from ModBase that were used in TB-drugome. For each homology model, the ModBase model code is given, as well as the gene accession number, gene name and description of the M.tb protein. N.B. Further information about each homology model can be found on the ModBase website.
(0.24 MB XLS)

Table S6 Parameters to fit the power law distribution for drug connections in the TB-drugome.
(0.03 MB DOC)

Table S7 Parameters to fit the power law distribution for target connections in the TB-drugome derived from the fraction of structurally characterized drugs.
(0.03 MB DOC)

Table S8 Parameters to fit the power law distribution for drug connections in the TB-drugome derived from the fraction of structurally characterized drugs.
(0.03 MB DOC)
