# Supplementary material for: The Mycobacterium tuberculosis Drugome and Its Polypharmacological Implications
Source: PLoS Comput Biol. 2010 Nov 4;6(11):e1000976. doi: 10.1371/journal.pcbi.1000976 (PMC2973814; doi:10.1371/journal.pcbi.1000976)
Supplement: Figure S1 — Fitting of the distribution of drug connections to a power-law distribution for co-crystallized drug complexes in the PDB. (0.15 MB DOC) [file pcbi.1000976.s001.doc]

**
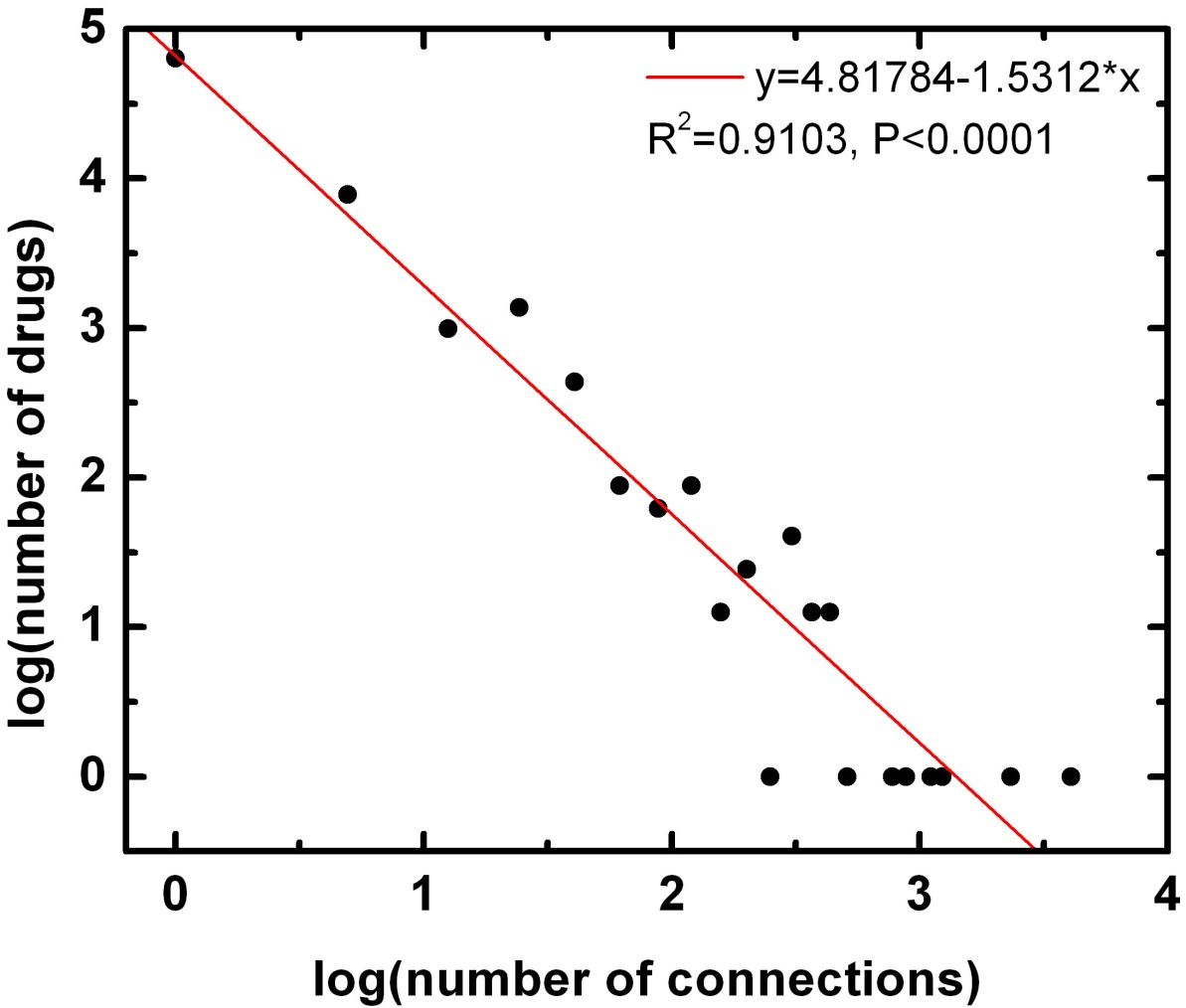
**

Figure S1. Fitting of the distribution of drug connections to a power-law distribution for co-crystallized drug complexes in the PDB.
