# Supplementary material for: The Mycobacterium tuberculosis Drugome and Its Polypharmacological Implications
Source: PLoS Comput Biol. 2010 Nov 4;6(11):e1000976. doi: 10.1371/journal.pcbi.1000976 (PMC2973814; doi:10.1371/journal.pcbi.1000976)
Supplement: Figure S2 — Fitting of the distribution of drug connections to a power-law distribution for the TB-drugome and a random network. (1.06 MB DOC) [file pcbi.1000976.s002.doc]

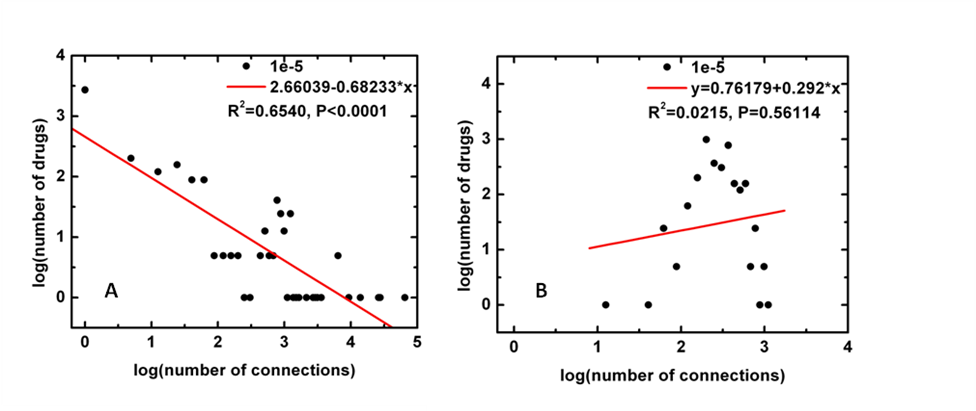


Figure S2. Fitting of the distribution of drug connections to a power-law distribution for (A) the TB-drugome and (B) a random network. A SMAP *P*-value threshold of 1.0e-5 was used.
