# Supplementary material for: The Mycobacterium tuberculosis Drugome and Its Polypharmacological Implications
Source: PLoS Comput Biol. 2010 Nov 4;6(11):e1000976. doi: 10.1371/journal.pcbi.1000976 (PMC2973814; doi:10.1371/journal.pcbi.1000976)
Supplement: Figure S3 — Fraction of the largest connected component (nLCC) in the network for the TB-drugome and a random network at different SMAP P-value thresholds. (0.05 MB DOC) [file pcbi.1000976.s003.doc]

**
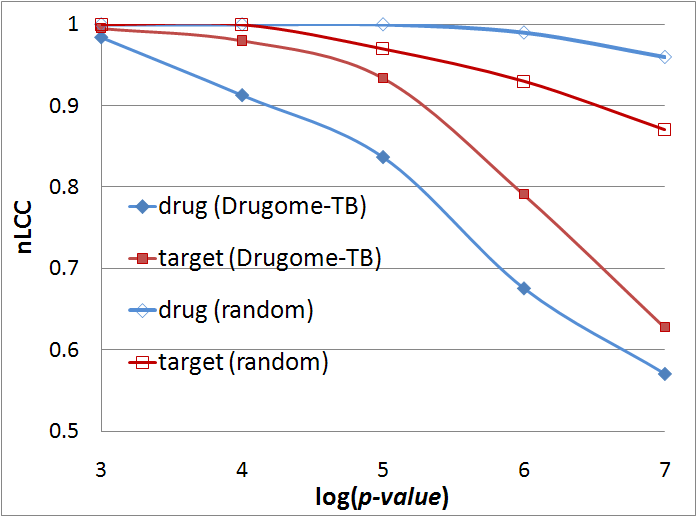
**

**Figure S3. Fraction of the largest connected component (nLCC) in the network for the TB-drugome and a random network at different SMAP *P*-value thresholds.**
