# Supplementary material for: The Mycobacterium tuberculosis Drugome and Its Polypharmacological Implications
Source: PLoS Comput Biol. 2010 Nov 4;6(11):e1000976. doi: 10.1371/journal.pcbi.1000976 (PMC2973814; doi:10.1371/journal.pcbi.1000976)
Supplement: Figure S4 — The clustering coefficient of the TB-drugome derived from different fractions of structurally characterized drugs. (0.07 MB DOC) [file pcbi.1000976.s004.doc]

**
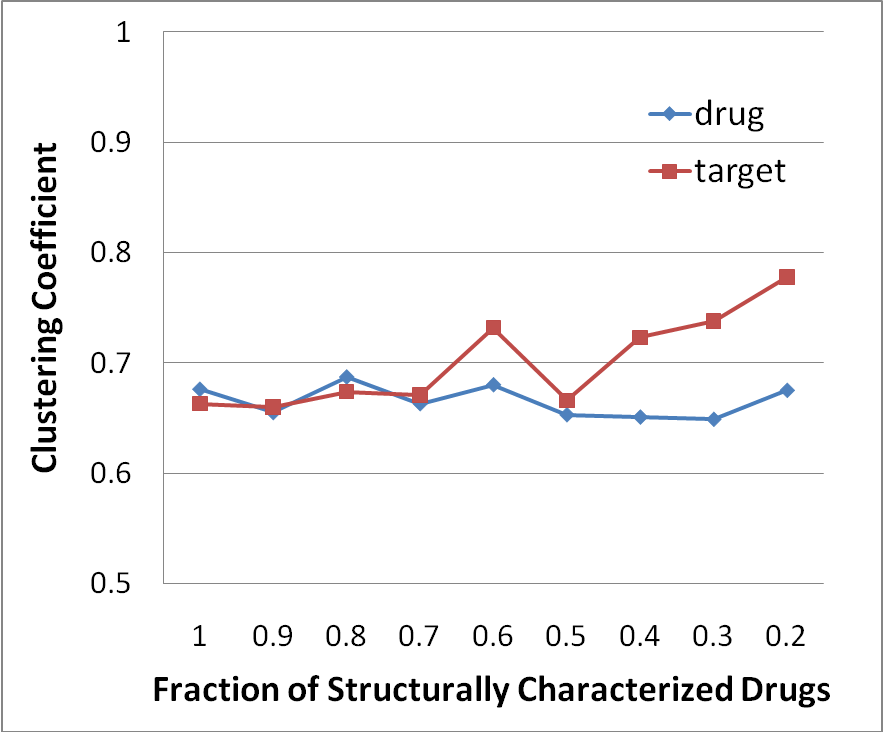
**

**Figure S4. The clustering coefficient of the TB-drugome derived from different fractions of structurally characterized drugs.**
