# Supplementary material for: The Mycobacterium tuberculosis Drugome and Its Polypharmacological Implications
Source: PLoS Comput Biol. 2010 Nov 4;6(11):e1000976. doi: 10.1371/journal.pcbi.1000976 (PMC2973814; doi:10.1371/journal.pcbi.1000976)
Supplement: Figure S5 — Predicted drug binding site and poses in adenylyl cyclase. (0.75 MB DOC) [file pcbi.1000976.s005.doc]

**
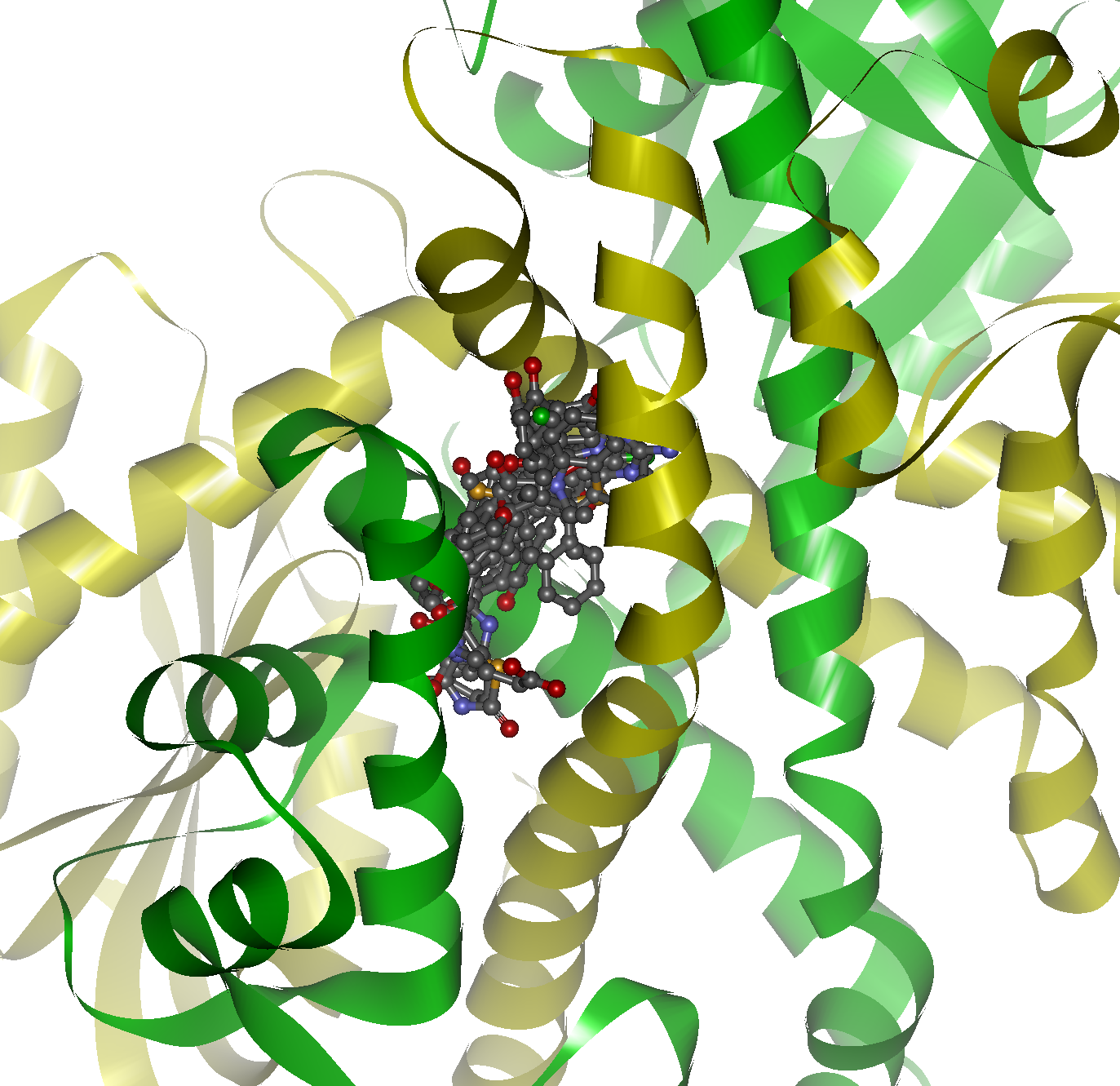
**

**Figure S5. Predicted drug binding site and poses in adenylyl cyclase. The protein is represented as a green ribbon model. Drugs are represented as ball-and-stick models. The atoms of C, O, N, and S are colored grey, red, blue and yellow, respectively.**
