# Supplementary material for: The Mycobacterium tuberculosis Drugome and Its Polypharmacological Implications
Source: PLoS Comput Biol. 2010 Nov 4;6(11):e1000976. doi: 10.1371/journal.pcbi.1000976 (PMC2973814; doi:10.1371/journal.pcbi.1000976)
Supplement: Table S6 — Parameters to fit the power law distribution for drug connections in the TB-drugome. (0.03 MB DOC) [file pcbi.1000976.s011.doc]

**Table S6: Parameters to fit the power law distribution for drug connections in the TB-drugome.**

| **SMAP P-value cutoff** | **k** | **log(a)** | **R^2^** | **P-value** |
| --- | --- | --- | --- | --- |
| 1e-3 | -0.33166 | 1.79364 | 0.5737 | < 0.0001 |
| 1e-4 | -0.43886 | 1.93342 | 0.5542 | < 0.0001 |
| 1e-5 | -0.68233 | 2.66039 | 0.6540 | < 0.0001 |
| 1e-6 | -0.96530 | 3.12986 | 0.7259 | < 0.0001 |
| 1e-7 | -1.12010 | 3.10865 | 0.7202 | <0.0001 |
