# Supplementary material for: The Mycobacterium tuberculosis Drugome and Its Polypharmacological Implications
Source: PLoS Comput Biol. 2010 Nov 4;6(11):e1000976. doi: 10.1371/journal.pcbi.1000976 (PMC2973814; doi:10.1371/journal.pcbi.1000976)
Supplement: Table S7 — Parameters to fit the power law distribution for target connections in the TB-drugome derived from the fraction of structurally characterized drugs. (0.03 MB DOC) [file pcbi.1000976.s012.doc]

**Table S7: Parameters to fit the power law distribution for target connections in the TB-drugome derived from the fraction of structurally characterized drugs (SMAP *P*-value = 1.0e-5).**

| **Fraction** | **k** | **log(a)** | **R^2^** | ***P*-value** |
| --- | --- | --- | --- | --- |
| 0.2 | -2.51402 | 5.31109 | 0.9705 | 0.00217 |
| 0.3 | -2.67072 | 5.57480 | 0.9038 | 0.00101 |
| 0.4 | -2.10370 | 5.29546 | 0.8970 | <0.0001 |
| 0.5 | -2.13167 | 5.65737 | 0.9277 | <0.0001 |
| 0.6 | -1.90097 | 5.65546 | 0.9692 | <0.0001 |
| 0.7 | -1.94752 | 5.67210 | 0.9099 | <0.0001 |
| 0.8 | -1.84491 | 5.64111 | 0.9477 | <0.0001 |
| 0.9 | -1.86857 | 5.71066 | 0.9351 | <0.0001 |
| 1.0 | -1.74780 | 5.65070 | 0.9436 | <0.0001 |
