# Supplementary material for: The Mycobacterium tuberculosis Drugome and Its Polypharmacological Implications
Source: PLoS Comput Biol. 2010 Nov 4;6(11):e1000976. doi: 10.1371/journal.pcbi.1000976 (PMC2973814; doi:10.1371/journal.pcbi.1000976)
Supplement: Table S8 — Parameters to fit the power law distribution for drug connections in the TB-drugome derived from the fraction of structurally characterized drugs. (0.03 MB DOC) [file pcbi.1000976.s013.doc]

**Table S8: Parameters to fit the power law distribution for drug connections in the TB-drugome derived from the fraction of structurally characterized drugs (SMAP *P*-value = 1.0e-5).**

| **Fraction** | **k** | **log(a)** | **R^2^** | ***P*-value** |
| --- | --- | --- | --- | --- |
| 0.2 | -0.28112 | 1.07900 | 0.6008 | 0.00113 |
| 0.3 | -0.40365 | 1.43842 | 0.5146 | 0.00119 |
| 0.4 | -0.53878 | 1.88273 | 0.6436 | <0.0001 |
| 0.5 | -0.50628 | 1.77101 | 0.5695 | <0.0001 |
| 0.6 | -0.47959 | 1.80075 | 0.4527 | <0.0001 |
| 0.7 | -0.57935 | 2.20623 | 0.5435 | <0.0001 |
| 0.8 | -0.59672 | 2.27544 | 0.5393 | <0.0001 |
| 0.9 | -0.65304 | 2.49195 | 0.5824 | <0.0001 |
| 1.0 | -0.68233 | 2.66039 | 0.6540 | < 0.0001 |
